# Non-Gaussianity of invariant measures to SPDEs in Da Prato–Debussche regime

**DOI:** 10.1007/s40072-025-00370-3

**Published:** 2025-06-19

**Authors:** Ajay Chandra, Ilya Chevyrev

**Affiliations:** 1 Imperial College London, London, England; 2 SISSA, Trieste, Italy

**Keywords:** Stochastic PDEs, Da Prato–Debussche, Invariant measure, Markov process, Euclidean field theory

## Abstract

We propose an elementary method to show non-Gaussianity of invariant measures of parabolic stochastic partial differential equations with polynomial non-linearities in the Da Prato–Debussche regime. The approach is essentially algebraic and involves using the generator equation of the SPDE at stationarity. Our results in particular cover the $\Phi ^4_\delta$ measures in dimensions $\delta <\frac{14}{5}$, which includes cases where the invariant measure is singular with respect to the invariant measure of the linear solution.

## Introduction

**Background.** There has been significant interest in recent years in studying Euclidean quantum field theories (EQFT) using methods from stochastic analysis. One of the most prominent of these methods is parabolic stochastic quantisation, wherein one studies an EQFT using its Langevin dynamic (or stochastic quantisation equation), an idea first proposed by Parisi–Wu [57].

By EQFT, we mean a Gibbs-type probability measure on $\mathcal {S}'({\textbf{R}}^d)$ given heuristically by

1.1 $$\begin{aligned} \mu (\,\textrm{d}u) \propto \exp (-S(u)) \mathcal {D}u \end{aligned}$$

where $\mathcal {D}u$ is a formal Lebesgue measure on $\mathcal {S}'({\textbf{R}}^d)$ and $S:\mathcal {S}'({\textbf{R}}^d)\rightarrow {\textbf{R}}$ is an action. The Langevin dynamic, in this case, is the stochastic (partial) differential equation

1.2 $$\begin{aligned} \partial _t u = -\frac{1}{2} \nabla S(u) + \xi \end{aligned}$$

where $\xi$ is a space-time white noise on ${\textbf{R}}\times {\textbf{R}}^d$ with respect to some metric on $\mathcal {S}'({\textbf{R}}^d)$ (we will always take the $L^2$ metric) and $\nabla S$ is the gradient of *S* with respect to the same metric.

Of course, both (1.1) and (1.2) are formal as written and giving rigorous meaning to them is often challenging. For example, the $\Phi ^{p+1}_d$ measures, for which $S(u) = \int _{{\textbf{R}}^d}\frac{1}{2}|\nabla u|^2 + \frac{1}{p+1}|u|^{p+1}$ with $p\ge 3$ an odd integer, were the subject of intensive investigation in the field of constructive quantum field theory, see e.g. [39]. Their construction in dimension $d\ge 2$ is non-trivial and requires a renormalisation of the Lagrangian density $\frac{1}{2}|\nabla u|^2 + \frac{1}{p+1}|u|^{p+1}$. For such EQFT’s, the Langevin dynamic (1.2) is then given by the SPDE

1.3 $$\begin{aligned} \partial _t u = \Delta u - u^p + \xi \;, \end{aligned}$$

whose solution theory, even locally-in-time, is non-trivial and requires a renormalisation of the equation for $d\ge 2$ due to the singularity of $u^p$.

A celebrated result of Da Prato–Debussche (DPD) [32] gave a rigorous meaning to so-called strong solutions to (1.3) on the 2-dimensional torus $\textbf{T}^2$ using smooth approximations. Their result can be approximately stated as follows: let $\xi ^\varepsilon$ be a smooth approximation of the white noise $\xi$ on ${\textbf{R}}\times \textbf{T}^2$ at scale $\varepsilon >0$. Then there exist constants $\sigma ^2_\varepsilon$ with $\lim _{\varepsilon \downarrow 0}\sigma ^2_\varepsilon = \infty$ such that the solution $u^\varepsilon :[0,\infty )\times \textbf{T}^2\rightarrow {\textbf{R}}$ to the regularised and renormalised SPDE

1.4 $$\begin{aligned} \partial _t u^\varepsilon = \Delta u^\varepsilon - H^{\sigma ^2_\varepsilon }_{p}(u^\varepsilon ) + \xi ^\varepsilon \end{aligned}$$

converges to a Markov process *u* globally in time for $\mu$-a.e. initial condition, where $H^{\sigma ^2_\varepsilon }_{p}$ is the *p*-th Hermite polynomial with variance $\sigma ^2_\varepsilon$ and $\mu$ is the $\Phi ^p_2$ measure on $\textbf{T}^2$, which is invariant for *u*. Their method is based on a simple yet very useful decomposition of the solution, which we review in Section 3. (The restriction to $\mu$-a.e. initial condition in [32] comes from the use of the invariant measure in the passage from local to global solutions. Later, [53] showed existence of global solutions for all initial conditions using only PDE arguments.)

Later on, several solution theories appeared that were able to solve (1.3) in dimension $d=3$ and even in fractional dimension $d<4$ (known as the subcritical or super-renormalisable regime), see [38, 44] and [5, 12, 13, 18, 34, 48, 49, 56] for a highly incomplete sample of further developments. These solution theories in large part extend rough path theory [50], designed to solve singular *ODEs*, to higher dimensions and an important step is to incorporate renormalisation like in (1.4), which is typically not present in the ODE setting (though see [6, 7, 42]). The method of DPD can be seen as an important precursor to these theories, and the threshold at which one can solve renormalised equations like (1.3)-(1.4) without appealing to rough path-type arguments is now sometimes called the ‘DPD regime’. We note that a similar method appeared for the stochastic Navier–Stokes equations [31] and that the DPD decomposition can also be useful for non-singular equations in obtaining a priori estimates, see e.g. [21].

An important motivation for analysing the Langevin dynamic (1.2) is its potential use in studying the measure (1.1). Indeed, the study of the dynamic has led to new constructions of the $\Phi ^4_d$ measures for $d<4$ [1, 24, 29, 35, 36, 52, 55], proofs of new tail bounds [47], and proof of exponential decay of correlations in exponential models [37]. The Langevin dynamic has also been studied for Yang–Mills–Higgs models in dimensions 2 and 3 [4, 14, 15, 26, 58], the sine-Gordon model [3, 17, 22, 46], and tensor field theories [16]. For other stochastic analytic approaches to EQFTs, see [10, 40].

**Our contribution.** The main purpose of this article is to present an elementary and, as far as we can tell, new method to prove non-Gaussianity of invariant measures of SPDEs like (1.3) in the DPD regime (including noises that are more singular than white noise on ${\textbf{R}}\times \textbf{T}^2$). Our method is essentially algebraic and uses the generator equation of the SPDE at stationarity, i.e. an infinite-dimensional Euler–Lagrange equation. This equation, as remarked in [11], is essentially an integrated, second order integration by parts (Dyson–Schwinger) identity (see Remark 2.6), but its use in the current context appears new.

We remark that, by a recent result of [45], the DPD regime, to which our method applies, is larger than the regime in which the invariant measure is absolutely continuous with respect to the Gaussian free field. (For this latter regime, proving non-Gaussianity is even simpler than our proposed method.) Furthermore, our method applies to polynomial equations with non-local terms such as the $\Phi ^3_2$ model, see Remarks 2.9 and 3.25 (see also the end of Section 3 for comments on the Sine-Gordon model).

Along the way, we review the local well-posedness result of DPD and make precise the form of the generator of *u*. There has been interest lately in constructing the generator for singular stochastic equations. For example, [41] constructs the generator for the stochastic Burgers equation. In comparison, the problem of constructing the generator for (1.3) in the DPD regime is much simpler but still has subtleties which we aim to clarify. For example, we need to view *u* as a Markov process with a state space of ‘rough distributions’, which has empty intersection with smooth functions, and this viewpoint is a bit different from other works we are aware of.

**Related works.** Non-Gaussianity is an important property of an EQFT as it is a necessary condition for the physical quantum field theory, obtained by going to Minkowski space from Euclidean space via Wick rotation, to be an interacting theory. Our result that the invariant measure of *u* is non-Gaussian is certainly not new. In the case of two dimensions, non-Gaussianity is an immediate consequence of the asymptotic moment formula obtained in [30], and the work [51] carries out the construction and proves non-Gaussianity in the harder three dimensional case. Closer to our setting is [9] which uses Dyson–Schwinger equations, in combination with skeleton inequalities, to give constructions and proofs of non-Gaussianity of the $\Phi ^4_2$ and $\Phi ^4_3$ measures for small coupling strengths. Our arguments, we believe, are more elementary but do not cover $\Phi ^4_3$ (but do apply to all coupling strengths).

Our use of the Langevin dynamic to prove non-Gaussianity is also not the first: [2, 36, 47] show non-Gaussianity of $\Phi ^4_3$ (at all coupling strengths) by using the dynamic, which is *outside* the DPD regime and is thus, in principle, more general. Our approach, however, is different and somewhat simpler, and demonstrates yet another use of the Langevin dynamic in the study of EQFTs.

**Article structure.** In Section 2 we demonstrate the idea of our method on a classically well-posed equation that does not require renormalisation, namely (1.3) posed on ${\textbf{R}}_+\times \textbf{T}$ and driven by white noise.

In Section 3, we turn to the DPD regime. In Section 3.1 we review the DPD argument, presenting a viewpoint that allows us to make precise the generator of the SPDE in Section 3.2. In Section 3.3 we use the generator to give an elementary proof of non-Gaussianity of the SPDEs under consideration.

Finally, in Appendix A, we show that the invariant measure of the SPDEs we consider are, in general, singular with respect to the invariant measure of the linear solution (i.e. the GFF).

### Notation

Let $\textbf{T}^d={\textbf{R}}^d/{\textbf{Z}}^d$ denote the torus. We will implicitly identify $\textbf{T}^d$ with $[-\frac{1}{2},\frac{1}{2})^d$ and identify functions on $\textbf{T}^d$ with periodic functions on ${\textbf{R}}^d$. We write ${\langle \xi ,f\rangle }$ for the pairing between a distribution $\xi$ and function *f*. We let $x\wedge y = \min \{x,y\}$.

We denote $X=O(Y)$ and $X\lesssim Y$ to mean that there exists $C>0$ such that $X\le CY$, where the dependencies of *C* are clear from the context.

We write $\mathcal{C}$ and $\mathcal{C}^0$ for the space of continuous functions and bounded measurable functions respectively. For $\alpha >0$, we let $\mathcal{C}^\alpha (\textbf{T}^d)$ denote the closure of smooth functions $f:\textbf{T}^d \rightarrow {\textbf{R}}$ under the usual norm

$$\begin{aligned} \Vert f\Vert _{\mathcal{C}^\alpha (\textbf{T}^d)} {\mathop {=}\limits ^{{\tiny def}}}\max _{|k|<\lfloor \alpha \rfloor } \Vert \partial ^k f\Vert _\infty + \max _{|k|=\lfloor \alpha \rfloor }\Vert \partial ^{k} f\Vert _{\mathcal{C}^{\alpha -\lfloor \alpha \rfloor }} \end{aligned}$$

where, for $$\eta \in [0,1]$$,

$$\begin{aligned} \Vert f\Vert _{\mathcal{C}^\eta } = \sup _{x\ne y} |x-y|^{-\eta }|f(x)-f(y)|\;, \end{aligned}$$

and where $\lfloor \alpha \rfloor$ is the floor of $\alpha$ and $\partial ^k f$ is the usual *k*-th derivative of *f* for a multi-index $k\in {\textbf{N}}^d$, ${\textbf{N}}= \{0,1,\ldots \}$, and where we denote $|k| = \sum _{i=1}^d k_i$. We similarly let $\mathcal{C}^k({\textbf{R}}^d)$ denote space of *k*-times continuously differentiable functions on ${\textbf{R}}^d$.

For $\mathfrak {K}\subset {\textbf{R}}\times \textbf{T}^d$, we make the same definition for $\mathcal{C}^\alpha (\mathfrak {K})$ except that, for a multi-index $k = (k_0,\ldots , k_d)\subset {\textbf{N}}^{1+d}$, we use the parabolic scaling $|k| = 2k_0 + \sum _{i=1}^d k_i$.

For $\alpha <0$, we write $\mathcal{C}^\alpha (\textbf{T}^d) \subset \mathcal{D}'(\textbf{T}^d)$ for the closure of smooth functions under the (inhomogenous) Hölder–Besov norm

$$\begin{aligned} \Vert \xi \Vert _{\mathcal{C}^\alpha (\textbf{T}^d)} = \sup _{x\in \textbf{T}^d}\sup _{\phi \in \mathcal{B}^r} \sup _{\lambda \in (0,1]} \lambda ^{-\alpha }|{\langle \xi ,\phi ^\lambda _x\rangle }|\;, \end{aligned}$$

where $r=-\lfloor \alpha \rfloor +1$, $\mathcal{B}^r$ is the set of all $\phi \in \mathcal{C}^\infty ({\textbf{R}}^d)$ with support in the ball $\{|z|<\frac{1}{4}\}$ and $\Vert \phi \Vert _{\mathcal{C}^r({\textbf{R}}^d)}\le 1$, and where $\phi ^\lambda _x\in \mathcal{C}^\infty (\textbf{T}^d)$ is given by $\phi ^\lambda _x (z) = \lambda ^{-d}\phi ((z-x)\lambda ^{-1})$. For $\mathfrak {K}\subset {\textbf{R}}\times \textbf{T}^d$, we define $\mathcal{C}^\alpha (\mathfrak {K})$ analogously except that the first supremum is over $x\in \mathfrak {K}$, and, in the second supremum, $\mathcal{B}^r$ is taken as the set of all $\phi \in \mathcal{C}^\infty ({\textbf{R}}\times {\textbf{R}}^d)$ with support in $\{|z|<\frac{1}{4}\}$ and $\Vert \phi \Vert _{\mathcal{C}^r({\textbf{R}}\times {\textbf{R}}^d)}\le 1$ and where we denote $\phi ^\lambda _x (z) = \lambda ^{-d-2}\phi ((z_0-x_0)\lambda ^{-2},(\tilde{z}-\tilde{x})\lambda ^{-1})$ and $z= (z_0,\tilde{z})\in {\textbf{R}}\times \textbf{T}^d$. For any $\alpha \in {\textbf{R}}$, if no domain is specified, we let $\mathcal{C}^\alpha$ denote $\mathcal{C}^{\alpha }(\textbf{T}^d)$.

For a random variable *X*, we write $X\sim \mu$ to mean that the law of *X* is $\mu$.

## Non-triviality in the classical case

To demonstrate the idea, we use the dynamic to show that invariant measures of a class of classically well-posed SPDEs are non-Gaussian. The basic example to keep in mind is $\Phi ^{p+1}_1$ for $p\ge 3$ an odd integer.

We fix space dimension $d=1$ in this section. We use the shorthand $O = (-1,2)\times \textbf{T}\subset {\textbf{R}}\times \textbf{T}$. Consider the classically well-posed stochastic PDE

2.1 $$\begin{aligned} (\partial _t -\Delta ) u = P(u) + \xi \;, \qquad u_0\in \mathcal{C}(\textbf{T})\;, \end{aligned}$$

where *P* is, for now, any function $P:\mathcal{C}(\textbf{T})\rightarrow \mathcal{C}(\textbf{T})$ for which there exists $\ell >0$ such that, for all $x,y\in \mathcal{C}(\textbf{T})$,

2.2 $$\begin{aligned} \Vert P(x)-P(y)\Vert _{\infty } \le \Vert x-y\Vert _\infty (2+\Vert x\Vert _\infty +\Vert y\Vert _\infty )^\ell \;. \end{aligned}$$

Furthermore, $\xi$ is a stationary space-time Gaussian distribution with covariance $\delta _0 \otimes \textrm{Cov}_{\xi } \in \mathcal {S}'({\textbf{R}}\times \textbf{T})$ for some $\textrm{Cov}_{\xi } \in \mathcal {S}'(\textbf{T})$ such that convolution with $\textrm{Cov}_{\xi }$ can be extended^1^to a bounded operator $L^2(\textbf{T}) \rightarrow L^2(\textbf{T})$. Note that $\xi$ is white in time and $\textrm{Cov}_\xi$ refers only to the *spatial* covariance of $\xi$. Moreover, by a Kolmogorov-type argument (see e.g. [27, Thm. 2.7]), for all $\alpha <\frac{1}{2}$ and $p\in [1,\infty )$,

2.3 $$\begin{aligned} {\textbf{E}}\Vert \xi \Vert _{\mathcal{C}^{\alpha -2}(O)}^p < \infty \;. \end{aligned}$$

The local Lipschtiz bound (2.2) readily implies the following deterministic local well-posedness result.

### Proposition 2.1

Suppose $\alpha \in (0,\frac{1}{2})$ and $\xi \in \mathcal{C}^{\alpha -2}(O)$ and $u_0\in \mathcal{C}(\textbf{T})$. Then there exists $\kappa >0$, depending only on $\alpha$, such that, for $T = (2+\Vert u_0\Vert _{\infty }+ \Vert \xi \Vert _{\mathcal{C}^{\alpha -2}(O)})^{-1/\kappa } \in (0,1)$, (2.1) admits a unique solution $u \in \mathcal{C}([0,T],\mathcal{C}(\textbf{T}))$ and

$$\begin{aligned} \sup _{s\in [0,T]}\Vert u_s\Vert _{\infty } \le 2\Vert u_0\Vert _{\infty }+1\;. \end{aligned}$$

We further make use of the following Assumption 2.2 on *global* solutions to (2.1).

### Assumption 2.2

For every $u_0\in \mathcal{C}(\textbf{T})$ and $\xi \in \mathcal{C}^{\alpha -2}(O)$ with $\alpha \in (0,\frac{1}{2})$, (2.1) admits a unique solution in $\mathcal{C}([0,1],\mathcal{C}(\textbf{T}))$ and there exists $m>0$ such that, for all $t\in (0,1]$,

2.4 $$\begin{aligned} \Vert u_t\Vert _\infty \le (2+ \Vert u_0\Vert _\infty + \Vert \xi \Vert _{\mathcal{C}^{\alpha -2}(O)} + t^{-1})^m\;. \end{aligned}$$

### Remark 2.3

If *P* is a polynomial of degree $p\ge 2$, then Assumption 2.2 is equivalent to *p* being odd and that the leading coefficient of *P* is negative, i.e. $P(x)= \theta x^p + \sum _{i=0}^{p-1} a_ix^i$ with $\theta <0$ and $a_i\in {\textbf{R}}$. In this case, one moreover has the stronger bound

$$\begin{aligned} \Vert u_t\Vert _\infty \le (2+ \Vert \xi \Vert _{\mathcal{C}^{\alpha -2}(O)} + t^{-1})^m\;, \end{aligned}$$

see e.g. [54, 55] where this bound is shown in similar situations.

By Proposition 2.1 and (2.3), (2.1) admits local-in-time solutions almost surely, which, under Assumption 2.2, are global.

### Lemma 2.4

(Generator) Suppose Assumption 2.2 holds. Let $u_0 \in \mathcal{C}(\textbf{T})$ and *u* solve (2.1). Consider $\phi _1,\ldots , \phi _k \in \mathcal{C}^5(\textbf{T})$ and $F \in \mathcal{C}^3({\textbf{R}}^k)$ such that the third derivative of *F* has at most polynomial growth. Denote

$$\begin{aligned} X_t {\mathop {=}\limits ^{{\tiny def}}}({\langle u_t,\phi _1\rangle }, \ldots , {\langle u_t,\phi _k\rangle }) \in {\textbf{R}}^k\;. \end{aligned}$$

Then

2.5 $$\begin{aligned} \lim _{t\downarrow 0}t^{-1}{\textbf{E}}[F(X_t) - F(X_0)] = \mathcal {L}F(u_0) \end{aligned}$$

where

$$\begin{aligned} \mathcal {L}F(u_0)  &   = \sum _{i=1}^k \partial _i F(X_0)({\langle u_0, \Delta \phi _i \rangle } + {\langle P(u_0),\phi _i\rangle }) \\  &   \quad + \frac{1}{2}\sum _{i,j=1}^k \partial _{i,j}^2 F(X_0) {\langle \textrm{Cov}_{\xi }*\phi _i,\phi _j\rangle }\;. \end{aligned}$$

Furthermore, there exists $q>0$, depending only on *F* and *P*, such that, for all $t\in (0,1)$,

2.6 $$\begin{aligned} |t^{-1}{\textbf{E}}[F(X_t) - F(X_0)]| \le (2+\Vert u_0\Vert _\infty )^q\;. \end{aligned}$$

### Proof

Throughout the proof, $q>0$ denotes a sufficiently large number depending only on *F* and *P*, whose value may change from line to line.

We can assume $\Vert \phi _i\Vert _{\mathcal{C}^5}\le 1$. Equipping ${\textbf{R}}^k$ with the norm $|z| = \max _{i=1,\ldots ,k} |z_i|$, we have $|X_t| \le \Vert u_t\Vert _\infty$. Therefore, by Taylor’s theorem,

2.7 $$\begin{aligned} F(X_t) - F(X_0)  &   = \sum _{i=1}^k \partial _iF(X_0) {\langle u_t-u_0,\phi _i\rangle } \nonumber \\  &   \quad + \frac{1}{2}\sum _{i,j=1}^k \partial _{i,j}^2 F(X_0) {\langle u_t-u_0,\phi _i\rangle }{\langle u_t-u_0,\phi _j\rangle } \nonumber \\  &   \quad + \sum _{i=1}^k O(K|{\langle u_t-u_0,\phi _i\rangle }|^3)\;, \end{aligned}$$

where $K = \sup \{|F^{(3)}(x)|\,:\, |x| \le \sup _{s\in [0,t]}\Vert u_s\Vert _\infty \}$ and $F^{(3)}$ is the third derivative of *F*. Note that $|\partial _i F(X_0)| + |\partial _{i,j}^2 F(X_0)| \le (2+\Vert u_0\Vert _\infty )^q$ since we assumed $F^{(3)}$ has polynomial growth.

We next compute the expectation of the right-hand side of (2.7). We write

$$\begin{aligned} u_t-u_0 = e^{t\Delta } u_0 - u_0 + \int _0^t e^{(t-s)\Delta } P(u_s)\,\textrm{d}s + \Psi _t \end{aligned}$$

where $\Psi _t = \int _0^t e^{(t-s)\Delta } \xi _s \,\textrm{d}s$. For the linear terms, since $\Vert e^{t\Delta }\phi _i - \phi _i - t\Delta \phi _i \Vert _\infty = O(t^2)$ due to $\phi _i \in \mathcal{C}^5$,

$$\begin{aligned} \Gamma _{1,i}{\mathop {=}\limits ^{{\tiny def}}}{\langle e^{t\Delta }u_0 - u_0, \phi _i\rangle } = t{\langle u_0,\Delta \phi _i\rangle } + O(t^2 \Vert u_0\Vert _\infty ) \end{aligned}$$

and

$$\begin{aligned} \Gamma _{2,i} {\mathop {=}\limits ^{{\tiny def}}}{\langle \int _0^t e^{(t-s)\Delta } P(u_s)\,\textrm{d}s,\phi _i\rangle }  &   = {\langle \int _0^t e^{(t-s)\Delta } P(u_0)\,\textrm{d}s, \phi _i \rangle } \\  &   \qquad + O\Big (t\sup _{s\in [0,t]}\Vert P(u_s)-P(u_0)\Vert _\infty \Big )\ \;. \end{aligned}$$

We also have

$$\begin{aligned} {\langle \int _0^t e^{(t-s)\Delta } P(u_0)\,\textrm{d}s, \phi _i \rangle }  &   = {\langle P(u_0) , \int _0^t e^{(t-s)\Delta } \phi _i\,\textrm{d}s \rangle }\\  &   \quad = {\langle tP(u_0), \phi _i \rangle } + {\langle P(u_0) , \int _0^t (e^{(t-s)\Delta } \phi _i - \phi _{i}) \,\textrm{d}s \rangle }\\  &   \quad = {\langle tP(u_0), \phi _i \rangle } + O(t^2 \Vert P(u_0)\Vert _\infty )\;. \end{aligned}$$

Moreover, since $\xi$ has zero expectation, ${\textbf{E}}{\langle \Psi _t,\phi _i\rangle } = 0$.

Next, for the quadratic terms,

$$\begin{aligned} {\textbf{E}}{\langle u_t-u_0,\phi _i\rangle }{\langle u_t-u_0,\phi _j\rangle }  &   = {\textbf{E}}{\langle \Psi _t , \phi _i\rangle } {\langle \Psi _t , \phi _j\rangle } + \Gamma _{3}\;. \end{aligned}$$

where

$$\begin{aligned} {\textbf{E}}{\langle \Psi _t , \phi _i\rangle } {\langle \Psi _t , \phi _j\rangle }  &   = \int _0^t {\langle \textrm{Cov}_\xi * e^{(t-s)\Delta }\phi _i, e^{(t-s)\Delta }\phi _j\rangle }\,\textrm{d}s \\  &   = t {\langle \textrm{Cov}_\xi * \phi _i, \phi _j\rangle } + O(t^2) \end{aligned}$$

and the ‘error term’ $\Gamma _3$, by Cauchy–Schwarz, is bounded above by a multiple of

$$\begin{aligned} \sum _{m=1}^k \sqrt{t} \sqrt{{\textbf{E}}(\Gamma _{1,m}^2 + \Gamma _{2,m}^2)} + {\textbf{E}}(\Gamma _{1,m}^2 + \Gamma _{2,m}^2)\;. \end{aligned}$$

To conclude the proof, it remains to bound the stochastic term $\sup _{s\in [0,t]}\Vert P(u_s)-P(u_0)\Vert _\infty$ in the definition of $\Gamma _{2,i}$ as well as the expectation of the error term $K |{\langle u_t-u_0,\phi _i\rangle }|^3$ in (2.7).

Define $\tau \in (0,\frac{1}{2})$ by $\tau ^{-1} = (2 + \Vert \xi \Vert _{\mathcal{C}^{\alpha -2}(O)} + \Vert u_0\Vert _\infty )^{-1/\kappa }$, where $\kappa ,\alpha >0$ are as in Proposition 2.1. Then, by Proposition 2.1,

$$\begin{aligned} \sup _{s\in [0,\tau ]}\Vert u_s\Vert _\infty \le 2\Vert u_0\Vert + 1\;, \end{aligned}$$

while by (2.4) in Assumption (2.2)

$$\begin{aligned} \sup _{s\in [\tau ,1]}\Vert u_s\Vert _\infty \le (2+ \Vert u_0\Vert _\infty + \Vert \xi \Vert _{\mathcal{C}^{\alpha -2}(O)} + \tau ^{-1})^m \lesssim \tau ^{-q}\;. \end{aligned}$$

By the moment bounds (2.3), for any $p\ge 1$, there exists $q>0$ such that ${\textbf{E}}\tau ^{-p} \le (2 + \Vert u_0\Vert _\infty )^q$, so in conclusion,

2.8 $$\begin{aligned} {\textbf{E}}\sup _{s\in [0,1]} \Vert P(u_s)\Vert _\infty ^2 \le (2 + \Vert u_0\Vert _\infty )^q\;. \end{aligned}$$

In particular, by dominated convergence, as $t\downarrow 0$,

$$\begin{aligned} {\textbf{E}}\sup _{s\in [0,t]}\Vert P(u_s)-P(u_0)\Vert _\infty ^2 \rightarrow 0 \end{aligned}$$

and the left-hand side is bounded by $(2 + \Vert u_0\Vert _\infty )^q$ for all $t\in [0,1]$.

Finally, by similar reasoning, using the fact that $F^{(3)}$ has polynomial growth, we have ${\textbf{E}}K |{\langle u_t-u_0,\phi _i\rangle }|^3 \le t^{3/2}(2+\Vert u_0\Vert _\infty )^q$. $\square$

### Lemma 2.5

Suppose Assumption 2.2 holds and that $\mu$ is a probability measure on $\mathcal{C}(\textbf{T})$ that is invariant for the dynamic (2.1). Suppose ${\textbf{E}}_{u_0\sim \mu }\Vert u_0\Vert ^q_\infty <\infty$ for all $q \in [1,\infty )$. Then, in the setting of Lemma 2.4,

2.9 $$\begin{aligned} {\textbf{E}}_{u_0\sim \mu } [ \mathcal {L}F(u_0)] =0\;. \end{aligned}$$

In particular, for $\phi \in \mathcal{C}^\infty (\textbf{T})$ and integer $k \ge 1$,

2.10 $$\begin{aligned}  &   {\textbf{E}}_{u_0\sim \mu } \Big [ k{\langle u_0, \phi \rangle }^{k-1}({\langle u_0, \Delta \phi \rangle }+ {\langle P(u_0),\phi \rangle }) \nonumber \\  &   \quad + \frac{k(k-1)}{2} {\langle u_0, \phi \rangle }^{k-2} {\langle \textrm{Cov}_\xi *\phi , \phi \rangle } \Big ] = 0\;, \end{aligned}$$

where the final term in the expectation is zero if $k=1$.

### Proof

For the first claim,

$$\begin{aligned} {\textbf{E}}_{u_0\sim \mu }[\mathcal {L}F(u_0)]  &   = {\textbf{E}}_{u_0\sim \mu } \Big [ \lim _{t\downarrow 0}t^{-1}{\textbf{E}}_{\xi }[F(X_t) - F(X_0)] \Big ] \\  &   \quad = \lim _{t\downarrow 0}{\textbf{E}}_{u_0\sim \mu }[t^{-1}{\textbf{E}}_{\xi }[F(X_t) - F(X_0)] =0\;, \end{aligned}$$

where the first equality is due to (2.5), the second equality is due to (2.6) and dominated convergence, and the final equality uses invariance of $\mu$ which implies ${\textbf{E}}_{u_0\sim \mu } {\textbf{E}}_{\xi } F(X_t) = {\textbf{E}}_{u_0\sim \mu } F(X_0)$. (2.9) follows by specialising to $F(x) = x^k$. $\square$

### Remark 2.6

Formally, for $\textrm{Cov}_{\xi } = \delta$ (i.e. white noise case) the generator equation (2.9) can also be derived from the Dyson–Schwinger equations, i.e. integration by parts. The latter in this context is

$$\begin{aligned} {\textbf{E}}\nabla f(u) = {\textbf{E}}f(u)(P(u) + \Delta u) \end{aligned}$$

for any (possibly field-valued) function *f*(*u*) and where $\nabla f(u)$ is the gradient of *f*, namely ${\langle \nabla f(u),v\rangle } = Df(u)(v)$. Taking $f = \nabla F(u)$ where $F(u) = F(X)$ and $X=({\langle u,\phi _1\rangle },\ldots ,{\langle u,\phi _k\rangle })$ and thus $\nabla F(u) = \sum _{i=1}^k \partial _i F(X)\phi _i$, we obtain

$$\begin{aligned} {\textbf{E}}\sum _{i,j} \partial _{i,j}^2 F(X) \phi _i\otimes \phi _j = {\textbf{E}}\sum _i \partial _i F(X) \phi _i\otimes (P(u) + \Delta u)\;. \end{aligned}$$

We then obtain the above generator by evaluating the (tensor) field on the diagonal $x=y$ and integrating over *x*. A similar derivation was noted in [11, Sec. 3].

### Theorem 2.7

Suppose that *P* is a polynomial of odd degree with negative leading coefficient. Then every invariant measure $\mu$ is not Gaussian.

### Example 2.8

The prototypical example to which the theorem applies is

$$\begin{aligned} (\partial _t -\Delta ) u = -u^p + \xi \;, \qquad u_0\in \mathcal{C}(\textbf{T})\;, \end{aligned}$$

which has invariant measure

$$\begin{aligned} \mu (\,\textrm{d}u) = Z^{-1} \exp \Big ( -\frac{1}{p+1}\int u^{p+1} \Big ) \nu (\,\textrm{d}u) \end{aligned}$$

where $\nu$ is the law of the Gaussian free field on $\textbf{T}$, i.e. periodic Brownian motion.

Before giving the proof of Theorem 2.7, we recall the definition of Hermite polynomials which will be used in this proof and also in Section 3.

Given any $\sigma \in {\textbf{R}}$, we define an associated sequence of Hermite polynomials $(H^{\sigma ^2}_{n})_{n \in {\textbf{N}}}$ on ${\textbf{R}}$ inductively, setting $H_{0}^{\sigma ^2}(x) = 1$ and, for $n > 0$,

2.11 $$\begin{aligned} H^{\sigma ^2}_{n}(x) = x H^{\sigma ^2}_{n-1}(x) - \sigma ^2 \frac{\textrm{d}}{\textrm{d}x} H^{\sigma ^2}_{n-1}(x)\;. \end{aligned}$$

### Proof of Theorem 2.7

By Remark 2.3, our condition on *P* implies both Assumption 2.2 and that $\sup _{u_0 \in \mathcal{C}}{\textbf{E}}\Vert u_1\Vert ^q_\infty <\infty$ for all $q \in [1,\infty )$. Taking $u_0\sim \mu$, we in particular have ${\textbf{E}}_{u_0}\Vert u_0\Vert ^q_\infty <\infty$. Therefore, by (2.10) of Lemma 2.5, for ${\langle \textrm{Cov}_\xi *\phi ,\phi \rangle }=1$ and $\phi \in \mathcal{C}^5(\textbf{T})$,

$$\begin{aligned} {\textbf{E}}\Big [k{\langle u_0,\varphi \rangle }^{k-1}({\langle u_0,\Delta \varphi \rangle } - {\langle P(u_0),\varphi \rangle }) + \frac{1}{2}k(k-1){\langle u_0,\varphi \rangle }^{k-2} \Big ] = 0\;. \end{aligned}$$

Using the shorthand $X = {\langle u_0,\varphi \rangle }$, $Y = {\langle P(u_0),\varphi \rangle }$, and $Z = {\langle u_0,\Delta \varphi \rangle }$, we can rewrite the above as

2.12 $$\begin{aligned} {\textbf{E}}{X^{k-1}(Y-Z)} = \frac{k-1}{2}{\textbf{E}}{X^{k-2}}\;,\quad \forall k \ge 1\;, \end{aligned}$$

where the right-hand side is understood as zero for $k=1$. It follows that, for every polynomial *Q*,

$$\begin{aligned} {\textbf{E}}Q(X)(Y-Z) = {\textbf{E}}Q'(X)/2\;. \end{aligned}$$

Hence ${\textbf{E}}Q(X-\mu )(Y-Z) = {\textbf{E}}Q'(X-\mu )/2$ for any $\mu \in {\textbf{R}}$. Taking $\mu ={\textbf{E}}X$ and denoting $\hat{X}{\mathop {=}\limits ^{{\tiny def}}}X-{\textbf{E}}X$, we obtain

$$\begin{aligned} {\textbf{E}}Q(\hat{X})(Y-Z) = {\textbf{E}}Q'(\hat{X})/2\;. \end{aligned}$$

Suppose now that $u_0$ is Gaussian with correlation function ${\textbf{E}}{u_0(x)u_0(y)} = C(x,y)$. Note that $\hat{X}$ is in the first homogeneous Wiener chaos. We treat *C* as a positive, bounded, symmetric operator on $L^2(\textbf{T})$ by $C\phi = \int C(x,\cdot )\phi (x) \,\textrm{d}x$. Since $\mu$ is a Gaussian measure on $\mathcal{C}(\textbf{T})$, we have ${\textbf{E}}\Vert u_0\Vert _\infty ^2<\infty$, hence $\sup _{x,y}|C(x,y)|<\infty$ and thus *C* is a bounded operator $L^1(\textbf{T})\rightarrow L^\infty (\textbf{T})$.

Taking $Q_k(x) = H^{\sigma ^2}_k(x)$ with $\sigma ^2 = {\textbf{E}}\hat{X}^2$, we claim that

2.13 $$\begin{aligned} {\textbf{E}}Q_k (\hat{X}) Y = 0 \quad \forall k\ge 2\;. \end{aligned}$$

Indeed, recall the recursion (2.11). It follows that, if $\sigma ^2 > 0$, then

$$\begin{aligned} {\textbf{E}}Q_k(\hat{X})(Y-Z) = {\textbf{E}}Q_{k}'(\hat{X})/2 \propto {\textbf{E}}\hat{X} Q_{k}(\hat{X}) - {\textbf{E}}Q_{k+1}(\hat{X})\;. \end{aligned}$$

By orthogonality of Hermite polynomials, the right-hand side vanishes for $k \in {\textbf{N}}\setminus \{1\}$. Since *Z* is in the 1st inhomogeneous Wiener chaos, we obtain (2.13). If, on the other hand, $\sigma ^2=0$, then $Q_k(\hat{X}) = 0$ almost surely for all $k\ge 1$, and we again obtain (2.13).

Note that $Q_k (\hat{X})$ has the Wiener-Itô decomposition

$$\begin{aligned} Q_k (\hat{X}) = I_k (\phi ^{\otimes k}) \end{aligned}$$

where $I_k :H^{\otimes _{s} k} \rightarrow L^2\mathbf {(P)}$ is the Wiener isometry, *H* is the Hilbert space given by completion of smooth functions with inner product ${\langle C \phi ,\psi \rangle }$, and $H^{\otimes _{s} k}$ is *k*-fold symmetric tensor product of *H*.

Recalling $Y=\int _{\textbf{T}} P(u_0)(x)\phi (x)\,\textrm{d}x$ and using our assumption that the leading term of *P*(*u*) is $\theta u^p$ for $\theta \ne 0$ and *p* odd, it readily follows that

2.14 $$\begin{aligned}  &   0 = {\textbf{E}}Q_p(\hat{X}) Y = \theta {\textbf{E}}I_{p}( \phi ^{ \otimes p} ) I_{p} \Big ( \int _{\textbf{T}} \delta ( \cdot - x)^{\otimes p} \phi (x) \,\textrm{d}x \Big ) \nonumber \\  &   q= \theta \langle \phi ^{ \otimes p} , \int _{\textbf{T}} \delta ( \cdot - x)^{\otimes p} \phi (x) \,\textrm{d}x \rangle _{H^{\otimes _{s} p}} = \theta \int _{\textbf{T}} (C\phi )(x)^{p} \phi (x) \,\textrm{d}x\;. \end{aligned}$$

Above, in the second equality we used the fact that only the top chaos part of *Y* contributes to the expectation which we then have written in terms of $I_{p}$, and in the third equality we used the Wiener isometry property of $I_p$.

Consider now $\phi \in L^2(\textbf{T})$ a non-zero real eigenfunction of *C* with eigenvalue $\lambda > 0$. Take $\phi _n\in \mathcal{C}^\infty (\textbf{T})$ such that $\phi _n\rightarrow \phi$ in $L^2$. Then, since $C:L^2\rightarrow L^\infty$ is bounded, we have $C\phi _n \rightarrow C\phi$ in $L^\infty$, hence

2.15 $$\begin{aligned} {\langle (C\phi _n)^p , \phi _n\rangle } \rightarrow {\langle (C\phi )^p,\phi \rangle } = \lambda ^p {\langle \phi ^p,\phi \rangle }\;, \end{aligned}$$

which is strictly positive since *p* is odd. But, due to (2.14) applied to $\phi _n$, ${\langle (C\phi _n)^p , \phi _n\rangle } = 0$, which is a contradiction. $\square$

### Remark 2.9

The argument above also works for the normalised $\Phi ^3_1$ model

$$\begin{aligned} (\partial _t -\Delta ) u = -u^2 - \Big (\int _{\textbf{T}} u^2\Big ) u + \xi \;, \qquad u_0\in \mathcal{C}(\textbf{T})\;, \end{aligned}$$

which has invariant measure

$$\begin{aligned} \mu (\,\textrm{d}u) = Z^{-1} \exp \Big ( -\frac{1}{3}\int _{\textbf{T}} u^{3} - \frac{1}{4}\Big (\int _{\textbf{T}} u^2\Big )^2 \Big ) \nu (\,\textrm{d}u) \end{aligned}$$

where $\nu$ is the law of the Gaussian free field on $\textbf{T}$.

More generally, we can allow *P*(*u*)(*x*) in (2.1) to be any polynomial with leading term

2.16 $$\begin{aligned} u(x)^\ell R\Big (\int _{\textbf{T}} u^2,\ldots , \int _{\textbf{T}} u^{2m}\Big )\;, \end{aligned}$$

where $\ell$ is odd and $R(y_1,\ldots , y_m)$ is a homogeneous polynomial which is non-zero whenever $y_1,\ldots , y_m > 0$.

In this case, the same argument would go through, except the integrand on the right-hand side of (2.14) would become

$$\begin{aligned} (C\phi )(x)^{\ell } R\Big (\int _{\textbf{T}} (C\phi )^2,\ldots , \int _{\textbf{T}} (C\phi )^{2m} \Big ) \phi (x) \end{aligned}$$

and one makes a corresponding change to (2.15) so that the final term becomes $\lambda ^p \int _{\textbf{T}} \phi ^{\ell +1} R\big (\int _{\textbf{T}} \phi ^2,\ldots , \int _{\textbf{T}} \phi ^{2m} \big ) \,\textrm{d}x$, which is non-zero by assumption.

## The Da Prato–Debussche regime

We now extend the argument in Section 2 to the DPD regime for equations of the form

3.1 $$\begin{aligned} (\partial _t +1 -\Delta ) u = P(u) + \xi \;,\quad u_0\in \mathcal{C}^\alpha (\textbf{T}^d)\;. \end{aligned}$$

Here and throughout this section, *P* is a polynomial of degree $p\ge 1$ and $\xi$ is a stationary Gaussian noise on ${\textbf{R}}\times \textbf{T}^d$ with covariance $\delta \otimes \textrm{Cov}_\xi \in \mathcal{D}'({\textbf{R}}\times \textbf{T}^d)$ (in particular white-in-time). We fix in this section the degree $p\ge 1$ and a regularity parameter $\alpha <0$.

In this section, we will progressively add assumptions on the polynomial *P* and the spatial covariance $\textrm{Cov}_\xi$ (see Assumptions 3.9, 3.14, 3.19, 3.21). We keep these assumptions separate to emphasise which properties of *P* and $\xi$ we are using at each step. However, the reader should keep in mind that they are all satisfied for $P(x) = \sum _{j=0}^p a_j x^j$ where *p* is odd and $a_p < 0$ and $|(1-\Delta )^{-1}*\textrm{Cov}_\xi (x)| \lesssim |x|^{\rho }$ with $\rho > \max \{-d/p, 2\alpha \}$ for $\alpha >2/(1-2p)$ (see Appendix A). In particular, our results apply to the $\Phi ^4_\delta$ model with $\delta <\frac{14}{5}$, see Example A.1.

### Remark 3.1

We consider $1-\Delta$ in (3.1) only for simplicity, and we could equally handle other elliptic operators like $(1-\Delta )^\sigma$ for $\sigma >0$.

### Da Prato–Debussche argument

We first recall the classical argument of Da Prato–Debussche [31, 32] for local well-posedness of stochastic PDEs of the form (3.1). There are several places where the argument is described, see e.g. [8, 19, 27, 43, 53, 59], and we do not claim significant novelty in our presentation or results in this subsection. (The only possible exception is Definition 3.11 which identifies a good state space for the Markov process associated to our SPDE.)

At this stage, we remark that, if $d=2$ and $\textrm{Cov}_{\xi }=\delta$ (the case considered in [32]), then by a Kolmogorov-type argument, $\xi$ is at best in $\mathcal{C}^{\alpha -2}((-1,2)\times \textbf{T}^d)$ for $\alpha <0$, hence, by parabolic regularity, *u* is at best in $\mathcal{C}^{\alpha }([0,1]\times \textbf{T}^d)$. The function *P*(*u*) is generally ill-defined on $\mathcal{C}^\alpha$ for $\alpha <0$ as there is no canonical way to extend the domain of the multiplication operator $(f,g)\mapsto fg$ from $\mathcal{C}\times \mathcal{C}$ to $\mathcal{C}^\alpha \times \mathcal{C}^\alpha$. The equation (3.1) thus appears ill-posed. Furthermore, replacing $\textrm{Cov}_{\xi }$ by an approximation of the identity $\delta ^\varepsilon$ and sending the mollification scale $\varepsilon \downarrow 0$ does not overcome this issue since, in general, the corresponding smooth solutions $u^\varepsilon$ will fail to converge to a non-trivial limit.

The idea in [32] is to consider the solution to the equation

3.2 $$\begin{aligned} (\partial _t +1 -\Delta )\Psi = \xi \end{aligned}$$

and decompose $u = v+\Psi$. Then the ‘remainder’ *v* solves the equation

3.3 $$\begin{aligned} (\partial _t +1 -\Delta ) v = P(v+\Psi )\;, \end{aligned}$$

where now we can hope to make sense of $P(v+\Psi )$ analytically provided that every power $\Psi ^k$ is replaced by another function $\Psi ^{:k:}$ that we can define stochastically.

#### Deterministic theory

Recall that we fixed $\alpha < 0$. Consider the Banach space of time-dependent distributions

$$\begin{aligned} \mathfrak {B}= \bigoplus _{k=1}^p \mathcal{C}([0,1],\mathcal{C}^{\alpha k}(\textbf{T}^d))\;. \end{aligned}$$

equipped with the norm

$$\begin{aligned} \Vert \boldsymbol{\Psi }\Vert _{\mathfrak {B}} = \max _{k=1,\ldots ,p} \sup _{t\in [0,1]}\Vert \Psi ^{(k)}_t\Vert _{\mathcal{C}^{\alpha k}(\textbf{T}^d)} \end{aligned}$$

where we denote a generic element of $\mathfrak {B}$ as $\boldsymbol{\Psi }= (\Psi ^{(1)},\ldots ,\Psi ^{(p)})$.

Instead of (3.3), we consider first the more general equation

3.4 $$\begin{aligned} (\partial _t +1 -\Delta ) v = \sum _{n=0}^p\sum _{k=0}^n a_{n,k} v^{n-k}\Psi ^{(k)} \end{aligned}$$

where $a_{n,k}\in {\textbf{R}}$. We suppose throughout the subsection that $\alpha (2p-1) > -2$, i.e.

3.5 $$\begin{aligned} 2+\alpha p > -\alpha (p-1)\;. \end{aligned}$$

Fix furthermore

3.6 $$\begin{aligned} \gamma \in (-\alpha (p-1),2+\alpha p)\;. \end{aligned}$$

(Note that $\gamma \in (0,2)$.) In particular, using that point-wise multiplication is a bounded bilinear map $\mathcal{C}^{\varsigma _1}(\textbf{T}^d) \times \mathcal{C}^{\varsigma _2}(\textbf{T}^d) \rightarrow \mathcal{C}^{\varsigma _1 \wedge \varsigma _2}(\textbf{T}^d)$ as long as $\varsigma _1+\varsigma _2 > 0$, our condition on $\gamma ,\alpha$ guarantees that multiplication is continuous as a map

3.7 $$\begin{aligned} \mathcal{C}^\gamma (\textbf{T}^d) \times \mathcal{C}^{\alpha k}(\textbf{T}^d) \rightarrow \mathcal{C}^{\alpha k}(\textbf{T}^d) \end{aligned}$$

for all $1\le k< p$. Consequently, the map

$$\begin{aligned} \mathcal{C}^\gamma (\textbf{T}^d)\times \mathcal{C}^{\alpha k}(\textbf{T}^d) \ni (v,\Psi ^{(k)}) \mapsto v^{n-k}\Psi ^{(k)} \in \mathcal{C}^{\alpha k}(\textbf{T}^d)\;, \end{aligned}$$

is locally Lipschitz for all $0\le k\le n\le p$. As a consequence, one can use the smoothing properties of the heat flow and Banach’s fixed point theorem to show the following local well-posedness for (3.4) (see [32, Prop. 4.4] and [59, Thm. 3.3] for similar statements).

##### Proposition 3.2

Consider $\gamma >0$ as in (3.6), $\eta \in ( -\frac{2}{p}, \gamma ]$, and $K > 2$. Denote

$$\begin{aligned} B_K {\mathop {=}\limits ^{{\tiny def}}}\{(v_0,\boldsymbol{\Psi }) \in \mathcal{C}^{\eta }(\textbf{T}^d)\times \mathfrak {B}\,:\, \Vert v_0\Vert _{\mathcal{C}^\eta (\textbf{T}^d)} + \Vert \boldsymbol{\Psi }\Vert _{\mathfrak {B}} \le K\}\;. \end{aligned}$$

Then there exists $\kappa >0$, depending only on $\alpha ,\eta ,p,\gamma$, such that, for $T = K^{-1/\kappa } \in (0,1)$, (3.4) admits a unique solution in the Banach space $\mathcal {S}$ of functions $v\in \mathcal{C}([0,T],\mathcal{C}^{\eta }(\textbf{T}^d))$ for which

3.8 $$\begin{aligned} \Vert v\Vert _{\mathcal {S}} {\mathop {=}\limits ^{{\tiny def}}}\sup _{t\in (0,T]} \{\Vert v_t\Vert _{\mathcal{C}^\eta (\textbf{T}^d)} + t^{-\frac{\eta \wedge 0}{2}}\Vert v_t\Vert _\infty + t^{\frac{\gamma -\eta }{2}} \Vert v_t\Vert _{\mathcal{C}^\gamma (\textbf{T}^d)}\} <\infty \;. \end{aligned}$$

Moreover, the solution map

$$\begin{aligned} B_K \ni (v_0,\boldsymbol{\Psi }) \mapsto v \in \mathcal {S}\end{aligned}$$

is Lipschitz and $\Vert v\Vert _{\mathcal {S}} \le C(\Vert v_0\Vert _{\mathcal{C}^\eta } + 1)$, where *C* depends only on $\alpha ,\eta ,p,\gamma$.

##### Remark 3.3

The threshold $\eta >-\frac{2}{p}$ is sharp and is necessary even for local well-posedness of $(\partial _t +1 -\Delta )v = \pm v^p$ with $v_0\in \mathcal{C}^{\eta }(\textbf{T}^d)$, see [20, 25].

We now return to our problem (3.3) and set $a_{n,k}$ such that

3.9 $$\begin{aligned} P(x+y) = \sum _{n=0}^p\sum _{k=0}^n a_{n,k} x^{n-k}y^k\;. \end{aligned}$$

In this case, if $\Psi ^{(k)}=\Psi ^k$, then (3.4) agrees with (3.3). However, for $\Psi \in \mathcal{C}^\alpha$ for $\alpha <0$, $\Psi ^k$ is generically not well-defined for $k\ge 2$, which is the whole reason for introducing the general equation (3.4).

We next introduce a set of distributions on which ‘Wick powers’ $\Psi ^{:k:}$ are well-defined. Fix a mollifier $\chi \in \mathcal{C}^\infty _c({\textbf{R}}^d)$ with $\int \chi = 1$, and write $\chi ^\varepsilon (x) = \varepsilon ^{-d}\chi (x/\varepsilon )$ and $\Psi ^\varepsilon = \Psi *\chi ^\varepsilon$.

##### Definition 3.4

Let $\mathfrak {A}\subset \mathcal{C}([0,1],\mathcal{C}^\alpha (\textbf{T}^d))$ be the subset of all time-dependent distributions $\Psi$ such that the collection of distributions

3.10 $$\begin{aligned} \boldsymbol{\Psi }= (\Psi ,\Psi ^{:2:},\ldots ,\Psi ^{:p:}) {\mathop {=}\limits ^{{\tiny def}}}\lim _{\varepsilon \downarrow 0} \big \{ H^{\sigma ^2_\varepsilon }_k(\Psi ^\varepsilon ) \big \}_{k=1,\ldots , p} \in \mathfrak {B}\end{aligned}$$

exists as a limit in $\mathfrak {B}$, where

3.11 $$\begin{aligned} \sigma ^2_\varepsilon = {\textbf{E}}_{\Phi \sim \nu }[ \Phi ^\varepsilon (0)^2] = {\langle \textrm{Cov}_{\xi } *(1-\Delta )^{-1}*\chi ^\varepsilon ,\chi ^\varepsilon \rangle } \end{aligned}$$

where $\nu$ is the law of the Gaussian field with covariance $\textrm{Cov}_{\xi }*(1-\Delta )^{-1}$.

##### Remark 3.5

The set $\mathfrak {A}$ is measurable (this follows, e.g. from the countable criterion for negative Hölder spaces of [28, Sec. 12] or [44, Prop. 3.20]). Furthermore, the map $\Psi \mapsto \Psi ^{:k:}$ is measurable.

It is not entirely trivial, however, that $\mathfrak {A}$ is non-empty. In fact, if $\lim _{\varepsilon \downarrow 0}\sigma _\varepsilon ^2 = \infty$, which is the case of interest in this section, then the intersection of $\mathfrak {A}$ with, say, $\mathcal{C}([0,1],L^\infty (\textbf{T}^d))$ is clearly empty. Propositions 3.10 and 3.12 will provide us with example distributions in $\mathfrak {A}$.

Recall the binomial identity for Hermite polynomials

3.12 $$\begin{aligned} H^{\sigma ^2}_k(x+y) = \sum _{j=0}^k \left( {\begin{array}{c}k\\ j\end{array}}\right) x^j H^{\sigma ^2}_{k-j}(y)\;. \end{aligned}$$

We further recall assumption (3.5) and a choice for $\gamma$ as in (3.6). The following result shows that $\mathfrak {A}$ is closed under addition of sufficiently regular functions.

##### Lemma 3.6

If $\Psi \in \mathfrak {A}$ and $v\in \mathcal{C}([0,1],\mathcal{C}^\gamma (\textbf{T}^d))$, then $\Psi +v\in \mathfrak {A}$ and

3.13 $$\begin{aligned} (v+\Psi )^{:k:} = \sum _{j=0}^k \left( {\begin{array}{c}k\\ j\end{array}}\right) v^j \Psi ^{:k-j:}\;. \end{aligned}$$

Moreover, for $\Psi$ fixed,

$$\begin{aligned} \mathcal{C}([0,1],\mathcal{C}^\gamma (\textbf{T}^d)) \ni v \mapsto (v+\Psi )^{:k:} \in \mathcal{C}([0,1],\mathcal{C}^{\alpha k}(\textbf{T}^d)) \end{aligned}$$

is locally Lipschitz and polynomial of degree *k*.

##### Proof

This follows from boundedness of multiplication (3.7), the fact that $\mathcal{C}^\gamma (\textbf{T}^d)$ is a Banach algebra, and the binomial identity (3.12). $\square$

##### Remark 3.7

The upper bound $\gamma <2+\alpha p$ in (3.6), which is assumed throughout this subsection, is not needed for Lemma 3.6.

For $\Psi \in \mathfrak {A}$ and a polynomial of one variable $P(t) = \sum _{j=0}^{p} a_{j} t^j$, we adopt the standard notation

$$\begin{aligned} \,:\!\! P(\Psi ) :\! = \sum _{j=0}^{p} a_{j} \Psi ^{:j:} \end{aligned}$$

for the corresponding local Wick polynomial.

For $\eta$ as in Proposition 3.2 and $\Psi \in \mathfrak {A}$, we now consider the ‘remainder equation’

3.14 $$\begin{aligned} (\partial _t +1 -\Delta ) v = \,:\!\! P(v+\Psi ) :\!\;,\quad v_0\in \mathcal{C}^{\eta }(\textbf{T}^d)\;, \end{aligned}$$

which, due to the binomial-type identity (3.13), is a special case of (3.4). Proposition 3.2 therefore implies that existence and uniqueness of local-in-time solutions for *v* in the Banach space $\mathcal {S}$.

##### Remark 3.8

By the local Lipschitz continuity of Proposition 3.2 in both the $\boldsymbol{\Psi }$ variable and the initial condition, the function $u = v+\Psi$ satisfies $u=\lim _{\varepsilon \downarrow 0}u^\varepsilon$, where $u^\varepsilon$ solves the ‘renormalised’ PDE

3.15 $$\begin{aligned} (\partial _t + 1 -\Delta ) u^\varepsilon = \sum _{j=0}^p a_j H_j^{\sigma _\varepsilon ^2}(u^\varepsilon ) + \xi *\chi ^\varepsilon \;,\quad u^\varepsilon _0 = v_0^\varepsilon + \Psi _0^\varepsilon \;, \end{aligned}$$

where $$\xi {\mathop {=}\limits ^{{\tiny def}}}(\partial _t +1-\Delta )\Psi $$.

#### Probabilistic theory

Our interest in $\mathfrak {A}$ comes from the fact that the random distribution $\Psi$ given as the solution to $(\partial _t +1-\Delta )\Psi = \xi$ with initial condition $\Psi _0\sim \nu$ for $\nu$ as in (3.11) almost surely takes values in $\mathfrak {A}$, i.e. the Wick powers of $\Psi$ are well defined and behave as required in Definition 3.4. We refer to the survey of Da Prato–Tubaro [33] for a more in-depth introduction to Wick powers.

Recall that $\alpha <0$ is fixed. As a warm up, we have the following result for the initial condition $\Psi _0$. Recall that $\nu$ is the Gaussian field with covariance $\textrm{Cov}_\nu = (1-\Delta )^{-1}*\textrm{Cov}_{\xi }$.

##### Assumption 3.9

There exists $\rho > \max \{-d/p,2\alpha \}$ such that $|\textrm{Cov}_\nu (x)|\lesssim |x|^{\rho }$ uniformly in $x\in \textbf{T}^d\setminus \{0\}$.

##### Proposition 3.10

Suppose Assumption 3.9 holds and denote $\Psi _0\sim \nu$. Then for all integers $k=1,\ldots ,p$ the limit $\lim _{\varepsilon \downarrow 0} H^{\sigma ^2_\varepsilon }_k(\Psi ^\varepsilon _0)$ exists in $\mathcal{C}^{\alpha k}(\textbf{T}^d)$ in $L^q(\nu )$ for all $q \in [1,\infty )$ and $\nu$-almost surely.

##### Proof

This is standard, so we only sketch the idea. Observe the bound

3.16 $$\begin{aligned} {\textbf{E}}{\langle \Psi ^{:k:}_0,\phi _x^\lambda \rangle }^2 = \int _{\textbf{T}^{2d}} \,\textrm{d}y \,\textrm{d}z \phi _x^\lambda (y)\phi _x^\lambda (z) \textrm{Cov}_{\nu }(y-z)^k \lesssim \lambda ^{\rho k}\;, \end{aligned}$$

where we used $\textrm{Cov}_{\nu }(y-z)^k \lesssim |y-z|^{\rho k}$ and $\rho k > -d$ by Assumption 3.9. The approximations $H^{\sigma ^2_\varepsilon }_k(\Psi ^\varepsilon _0)$ satisfy the same bound in which we replace $\textrm{Cov}_{\nu }$ by $\textrm{Cov}_{\nu }*\chi ^\varepsilon *\chi ^\varepsilon (-\cdot )$. Since $\alpha <\rho /2$, by equivalence of Gaussian moments and by a Kolmogorov-type argument, one can show that there exists $\kappa >0$ sufficiently small such that, for all $q\in [1,\infty )$, uniformly in $\varepsilon ,\bar{\varepsilon }\in (0,1)$,

$$\begin{aligned} {\textbf{E}}\Big [ \Vert H^{\sigma ^2_\varepsilon }_k(\Psi ^\varepsilon _0) - H^{\sigma ^2_{\bar{\varepsilon }}}_k(\Psi ^{\bar{\varepsilon }}_0) \Vert _{\mathcal{C}^{\alpha k}}^q \Big ]^{1/q}\lesssim |\varepsilon - \bar{\varepsilon }|^\kappa \;. \end{aligned}$$

Applying Kolmogorov’s theorem to the mollification variable $\varepsilon$, we conclude that, for all $q\in [1,\infty )$,

$$\begin{aligned} {\textbf{E}}\Big [ \Big (\sup _{0<\varepsilon< \bar{\varepsilon }< 1}|\varepsilon -\bar{\varepsilon }|^{-\kappa /2} \Vert H^{\sigma ^2_\varepsilon }_k(\Psi ^\varepsilon _0) - H^{\sigma ^2_{\bar{\varepsilon }}}_k(\Psi ^{\bar{\varepsilon }}_0) \Vert _{\mathcal{C}^{\alpha k}} \Big )^q \Big ] < \infty \;. \end{aligned}$$

This shows convergence of $H^{\sigma ^2_\varepsilon }_k(\Psi ^\varepsilon _0)$ in $\mathcal{C}^{\alpha k}$ in $L^q(\nu )$ and almost surely. $\square$

For the local solution theory of the *stochastic* PDE (3.1), we need to improve Proposition 3.10 and show the Wick powers of the linear solution $\Psi$ from (3.2) takes values in $\mathfrak {A}$. For our purposes, we crucially need the initial condition of $\Psi$ to be *any*^2^ element of a deterministic set $\mathfrak {C}\subset \mathcal{C}^\alpha (\textbf{T}^d)$ which supports $\nu$. The following definition of $\mathfrak {C}$ is the only result of this subsection that we believe is new.

##### Definition 3.11

Denote by $\mathfrak {C}$ the set of all $\Psi _0\in \mathcal{C}^\alpha (\textbf{T}^d)$ such that the stochastic process $\{H_k^{\sigma _\varepsilon ^2}(\Psi ^\varepsilon _t) \}_{t\in [0,1]}$ for $k=1,\ldots , p$, converges in $\mathcal{C}([0,1],\mathcal{C}^{\alpha k}(\textbf{T}^d))$ in $L^q(\P _\xi )$ for every $q\in [1,\infty )$ and $\P _\xi$-almost surely as $\varepsilon \downarrow 0$.

Above, as usual, $\Psi ^\varepsilon =\Psi *\chi ^\varepsilon$ and the stochastic process $\{\Psi _t\}_{t\in [0,1]}$ solves the linear stochastic heat equation $(\partial _t+1-\Delta )\Psi =\xi$ with initial condition $\Psi _0$, and $\xi$, as in (3.1), is Gaussian on ${\textbf{R}}\times \textbf{T}^d$ with covariance $\delta \otimes \textrm{Cov}_{\xi }$. We denote $\Psi ^{:k:} = \lim _{\varepsilon \downarrow 0}H_k^{\sigma _\varepsilon ^2}(\Psi ^\varepsilon )$ and3.17and, as earlier, $\boldsymbol{\Psi }= (\Psi ,\Psi ^{:2:},\ldots ,\Psi ^{:p:})$.

Elements of $\mathfrak {C}$ will play the role of sufficiently nice initial conditions for the SPDE (3.1) for which the generator exists (see Lemma 3.20). We first show that $\nu (\mathfrak {C})=1$.

##### Proposition 3.12

Suppose Assumption 3.9 holds and let $\Psi _0\sim \nu$. Then $\nu (\mathfrak {C})=1$ and  for all $q\in [1,\infty )$.

##### Proof

Denoting $Z^\varepsilon _t = H_k^{\sigma _\varepsilon ^2}(\Psi ^\varepsilon _t)$ and ${\textbf{E}}= {\textbf{E}}_{\Psi _0\sim \nu } {\textbf{E}}_\xi$, it suffices to prove that there exists $\kappa >0$ such that, for any $q\in [1,\infty )$, uniformly in $s,t\in [0,1], \varepsilon ,\bar{\varepsilon }\in (0,1)$,

3.18 $$\begin{aligned} ({\textbf{E}}\Vert Z^\varepsilon _{s} - Z^{\bar{\varepsilon }}_t\Vert _{\mathcal{C}^{\alpha k}(\textbf{T}^d)}^q )^{1/q} \lesssim |t-s|^{\kappa /2} + |\varepsilon -\bar{\varepsilon }|^\kappa \;. \end{aligned}$$

Indeed, denoting $Z^\varepsilon _{s,t} = Z^\varepsilon _t-Z^\varepsilon _s$, (3.18) implies that

$$\begin{aligned} ({\textbf{E}}\Vert Z^\varepsilon _{s,t} - Z^{\bar{\varepsilon }}_{s,t}\Vert _{\mathcal{C}^{\alpha k}(\textbf{T}^d)}^q )^{1/q} \lesssim |t-s|^{\kappa /4}|\varepsilon -\bar{\varepsilon }|^{\kappa /2} \end{aligned}$$

which in turn implies, by Kolmogorov’s theorem (first applied to the time variable *t* and then to the mollification scale $\varepsilon$), denoting $\mathcal {E}= \mathcal{C}^{\kappa /8}([0,1],\mathcal{C}^{\alpha k}(\textbf{T}^d))$,

$$\begin{aligned} {\textbf{E}}\Big [ \Big (\sup _{0<\varepsilon< \bar{\varepsilon }< 1}|\varepsilon -\bar{\varepsilon }|^{-\kappa /4} \Vert Z^\varepsilon - Z^{\bar{\varepsilon }} \Vert _{\mathcal {E}} \Big )^q \Big ] < \infty \;. \end{aligned}$$

In particular, sampling first $\Psi _0$, we have ${\textbf{E}}_\xi \sup _{\varepsilon ,\bar{\varepsilon }}(|\varepsilon -\bar{\varepsilon }|^{-\kappa /4}\Vert Z^\varepsilon -Z^{\bar{\varepsilon }}\Vert )^q = {\textbf{E}}[\sup _{\varepsilon ,\bar{\varepsilon }}(|\varepsilon -\bar{\varepsilon }|^{-\kappa /4}\Vert Z^\varepsilon - Z^{\bar{\varepsilon }} \Vert _{\mathcal {E}})^q|\Psi _0] \le C$ where *C* is a random variable in $L^1(\nu )$, which implies that, for $\nu$-almost every $\Psi _0$, the limit $\lim _{\varepsilon \downarrow 0}Z^\varepsilon$ exists in $\mathcal {E}$ in $L^q(\textbf{P} _\xi )$ and $\textbf{P}_\xi$-a.s., and this limit is in $L^q(\nu )$.

To prove (3.18), by equivalence of Gaussian moments and by decomposing a test function $\phi ^\lambda _x$ into a wavelet basis as in [44, Prop. 3.20], it furthermore suffices to show that

$$\begin{aligned} {\textbf{E}}|{\langle Z_{t}^{\varepsilon } -Z_{s}^{\bar{\varepsilon }},\phi ^\lambda _x\rangle }|^2 \lesssim \lambda ^{k\rho -\kappa }(|t-s|^{\kappa /2} + |\varepsilon -\bar{\varepsilon }|^\kappa ) \end{aligned}$$

uniformly over $x,\lambda ,\phi ,s,t,\varepsilon ,\bar{\varepsilon }$ - see the proof of [44, Prop. 9.5] for more detail. However, it is simple to verify that the (spatial) covariance of $\Psi _t$ is the same as that of $\Psi _0$. Therefore $Z_{t}^\varepsilon$ is the *k*-th Wick power of the stationary solution of the SHE driven by $\xi ^\varepsilon$, and the above bound follows from an identity similar to (3.16). $\square$

Next, we show that $\mathfrak {C}$ is stable under addition of sufficient regular functions.

##### Lemma 3.13

Consider $\Psi _0\in \mathfrak {C}$ and $v_0\in \mathcal{C}^\gamma (\textbf{T}^d)$ with $\gamma > -\alpha (p-1)$. Then $\Psi _0+v_0 \in \mathfrak {C}$ with .

##### Proof

This follows from Lemma 3.6 applied to $\Psi$ and the function $v_t = e^{t(\Delta -1)}v_0$, which is in $\mathcal{C}([0,1],\mathcal{C}^\beta (\textbf{T}^d))$ for any $\beta \in (-\alpha (p-1),\gamma )$ (this $\beta$ takes the role of $\gamma$ in Lemma 3.6; recall that no upper bound on $\gamma$ is required due to Remark 3.7). $\square$

For $\Psi _0\in \mathfrak {C}$, by definition, $\Psi \in \mathfrak {A}$ (i.e. $\boldsymbol{\Psi }\in \mathfrak {B}$) almost surely. Therefore, if (3.5) holds, then we can apply Proposition 3.2 to obtain local-in-time (random) solutions *v* to (3.14). We next consider an assumption that these solutions are *global*.

##### Assumption 3.14

(3.5) holds and, for $\eta ,\gamma$ as in Proposition 3.2 and all $\Psi \in \mathfrak {A}$ and $v_0\in \mathcal{C}^\eta (\textbf{T}^d)$, the equation (3.14) has a unique solution in $\mathcal{C}((0,1],\mathcal{C}^\gamma )$.

Since $\alpha >-\frac{2}{p}$ by (3.5), note that $\eta =\alpha$ satisfies the conditions of Proposition 3.2.

##### Definition 3.15

(Markov process) Suppose Assumption 3.14 holds. Consider $\gamma >0$. We define $u_t \in \mathcal{C}^\alpha$ with initial condition $u_0\in \mathcal{C}^{\alpha }$ as follows. Fix any $\Psi _0\in \mathfrak {C}$. Let $v\in \mathcal{C}((0,1],\mathcal{C}^\gamma )$ be the solution to (3.14) with initial condition $v_0 = u_0-\Psi _0\in \mathcal{C}^{\alpha }$. We then define $u_t = v_t+\Psi _t \in \mathcal{C}^\alpha$ for $t\in [0,1]$.

It may appear unnatural to make an arbitrary choice of $\Psi _0\in \mathfrak {C}$ in the definition of *u*. The next result implies that a different choice leads to the same definition of *u*.

##### Proposition 3.16

Suppose we are in the setting of Definition 3.15. Consider $\bar{\Psi }_0\in \mathfrak {C}$ and let $\bar{v}$ solve

$$\begin{aligned} (\partial _t +1 -\Delta )\bar{v} = \,:\!\! P(\bar{v}+\bar{\Psi }) :\!\;,\quad \bar{v}_0 = u_0 - \bar{\Psi }_0 \in \mathcal{C}^{\alpha }(\textbf{T}^d)\;, \end{aligned}$$

where $\bar{\Psi }$ solves $(\partial _t+1-\Delta )\bar{\Psi }= \xi$ with initial condition $\bar{\Psi }_0$ and where $\xi$ is the same noise appearing in the definition of $\Psi$. Then, almost surely, $\bar{v}_t + \bar{\Psi }_t = u_t$ for all $t\in [0,1]$.

##### Proof

This follows from Remark 3.8, which implies that *u* and $\bar{v}+\bar{\Psi }$ are both limits of (3.15). $\square$

The following result now shows that the solution *u* to (3.1) (in the sense of Definition 3.15) takes values in $\mathfrak {C}$.

##### Lemma 3.17

Suppose we are in the setting of Definition 3.15 and that Assumption 3.9 holds. Then, for every $t\in (0,1]$, $u_t \in \mathfrak {C}$ almost surely.

##### Proof

Let $\Psi$ and *v* be as in Definition 3.15 and fix $t\in (0,1]$. Then, almost surely, $v_t\in \mathcal{C}^\gamma (\textbf{T}^d)$. Furthermore, we write $\Psi _t = e^{t(\Delta -1)} \Psi _0 + \int _0^t e^{(t-s)(\Delta -1)}\xi _s\,\textrm{d}s$ wherein $e^{t(\Delta -1)} \Psi _0 \in \mathcal{C}^\infty (\textbf{T}^d)$. We also have $\int _0^t e^{(t-s)(\Delta -1)}\xi _s\,\textrm{d}s {\mathop {=}\limits ^{{\tiny law}}}\Phi + f$, where $\Phi \sim \nu$ and *f* is almost surely in $\mathcal{C}^\infty (\textbf{T}^d)$. By Proposition 3.12, $\Phi$ is almost surely in $\mathfrak {C}$. The conclusion now follows from Lemma 3.13. $\square$

##### Remark 3.18

Propositions 3.2 and 3.16 allow us to solve the $\Phi ^4_{\delta }$ SPDE for $\delta < 14/5$ (3.1) by constructing Wick powers of the linear solution $\Psi$ and deriving a well-posed equation for a remainder *v* with respect to this linear solution $\Psi$.

A conceptually similar but more involved argument allows us to go beyond $\delta < 14/5$ - if we solved for the higher order remainder $w_t = v_t - \int _0^t e^{(t-s)\Delta }\Psi ^{:p:}_s\,\textrm{d}s$ then we would obtain a solution theory for a broader range of parameters, e.g. $\Phi ^4_{\delta }$ for $\delta <3$. However, these sorts of methods cannot be pushed forward in a straightforward way to cross the $\delta = 3$ threshold.

### The Markov process

By Lemma 3.17, *u* from Definition 3.15 defines a Markov process on the set $\mathfrak {C}\subset \mathcal{C}^\alpha (\textbf{T}^d)$. Furthermore, for $u_0\in \mathfrak {C}$, we may simply take $\Psi _0 = u_0$ and thus $v_0=0$ in Definition 3.15. It is therefore natural to impose the following assumption on *P*, which is an analogue of Assumption 2.2.

#### Assumption 3.19

Assumption 3.14 holds and there exists $m>0$ such that, if $\Psi \in \mathfrak {A}$ and *v* is the solution to (3.14) with $v_0=0$, then for all $s\in (0,1]$,

3.19 $$\begin{aligned} \Vert v_s\Vert _{\mathcal{C}^\gamma (\textbf{T}^d)} \le (2+ \Vert \boldsymbol{\Psi }\Vert _{\mathfrak {B}} + s^{-1})^m\;. \end{aligned}$$

The following is an analogue of Lemma 2.4.

#### Lemma 3.20

Suppose Assumption 3.19 holds. Let $u_0 \in \mathfrak {C}$. Consider $\phi _1,\ldots , \phi _k \in \mathcal{C}^\infty (\textbf{T}^d)$ and $F \in \mathcal{C}^3({\textbf{R}}^k)$ with $F^{(3)}$ of polynomial growth. Denote

$$\begin{aligned} X_t {\mathop {=}\limits ^{{\tiny def}}}({\langle u_t,\phi _1\rangle }, \ldots , {\langle u_t,\phi _k\rangle }) \in {\textbf{R}}^k\;. \end{aligned}$$

Then

$$\begin{aligned} \lim _{t\rightarrow 0}t^{-1}{\textbf{E}}[F(X_t) - F(X_0)]= &   \sum _{i=1}^k \partial _i F(X_0)({\langle u_0, (\Delta -1)\phi _i \rangle } + {\langle \,:\!\! P(u_0) :\!,\phi _i\rangle }) \\  &   + \frac{1}{2}\sum _{i,j=1}^k \partial _{i,j} F(X_0) {\langle \textrm{Cov}_{\xi }*\phi _i,\phi _j\rangle }\;. \end{aligned}$$

and there exists $q>0$, depending only on *P* and *F*, such that, for all $t\in (0,\frac{1}{2})$,

#### Proof

We let $q>0$ denote a sufficiently large number, depending only on *F* and *P*, whose value may change from line to line. We write

$$\begin{aligned} u_t-u_0 = (e^{t(\Delta -1)}u_0 - u_0) +\int _0^t e^{(t-s)(\Delta -1)} \,:\!\! P( u_s ) :\!\,\textrm{d}s + \int _0^t e^{(t-s)(\Delta -1)} \xi _s \,\textrm{d}s\;. \end{aligned}$$

Arguing like in the proof of Lemma 2.4, for $\phi \in \mathcal{C}^\infty (\textbf{T}^d)$ we have

$$\begin{aligned} {\langle e^{t(\Delta -1)}u_0 - u_0,\phi \rangle } = t{\langle u_0,(\Delta -1)\phi \rangle } + O(t^2\Vert u_0\Vert _{\mathcal{C}^{\alpha }}) \end{aligned}$$

and

$$\begin{aligned}  &   \Big | {\langle \int _0^t e^{(t-s)(\Delta -1)} \,:\!\! P( u_s ) :\! \,\textrm{d}s,\phi \rangle } - t{\langle \,:\!\! P(u_0) :\!,\phi \rangle } \Big | \\  &   \qquad \lesssim t^2\Vert \,:\!\! P(u_0) :\!\Vert _{\mathcal{C}^{\alpha p}(\textbf{T}^d)} + t\sup _{s\in [0,t]} \Vert \,:\!\! P(u_s) :\! - \,:\!\! P(u_0) :\!\Vert _{\mathcal{C}^{\alpha p}(\textbf{T}^d)}\;. \end{aligned}$$

Let $\Psi _0=u_0$ and $v,\Psi$ be as in Definition 3.15, so that $u=v+\Psi$. By continuity of multiplication

3.20 $$\begin{aligned} \mathcal{C}^\gamma (\textbf{T}^d)\times \mathcal{C}^{\alpha j}(\textbf{T}^d) \ni (w,\psi ) \mapsto w\psi \in \mathcal{C}^{\alpha j}(\textbf{T}^d) \end{aligned}$$

and almost sure continuity of $[0,1] \ni t\mapsto (v_t,\Psi ^{:j:}_t) \in \mathcal{C}^\gamma (\textbf{T}^d)\times \mathcal{C}^{\alpha j}(\textbf{T}^d)$ for $j=0,\ldots ,p$, we have $\sup _{s\in [0,t]}\Vert \,:\!\! P(u_s) :\! - \,:\!\! P(u_0) :\!\Vert _{\mathcal{C}^{\alpha p}(\textbf{T}^d)} \rightarrow 0$ almost surely.

We next bound the moments of $\sup _{s\in [0,t]} \Vert \,:\!\! P(u_s) :\! \Vert _{\mathcal{C}^{\alpha p}(\textbf{T}^d)}$. Applying Proposition 3.2 with $\gamma = \eta$, there exists $\tau \in (0,\frac{1}{2}]$ such that $\tau ^{-1} \le (2+\Vert \boldsymbol{\Psi }\Vert _{\mathfrak {B}})^{q}$ and $\sup _{s\in [0,\tau ]}\Vert v_s\Vert _{\mathcal{C}^\gamma (\textbf{T}^d)} \le C$. Moreover, by (3.19) of Assumption 3.19, we have

$$\begin{aligned} \sup _{s\in [\tau ,1]}\Vert v_s\Vert _{\mathcal{C}^\gamma } \le (2+\Vert \boldsymbol{\Psi }\Vert _\mathfrak {B}+ \tau ^{-1})^m \le (2+\Vert \boldsymbol{\Psi }\Vert _\mathfrak {B})^q\;. \end{aligned}$$

Therefore . By continuity of multiplication (3.20) we thus have(which is analogous to the bound (2.8)). The rest of the proof follows in the same way as the proof of Lemma 2.4. $\square$

### Non-Gaussianity of the invariant measure

Suppose in the remainder of this section that we are in the setting of Definition 3.15. Let $\mu$ be a measure on $\mathcal{C}^\alpha (\textbf{T}^d)$ which is invariant for the Markov process $u_t$ from Definition 3.15. By Lemma 3.17, $\mu (\mathfrak {C}) = 1$.

#### Assumption 3.21

 for all $$q\in [1,\infty )$$.

We assume for the rest of this section that Assumptions 3.9, 3.19, and 3.21 hold.

#### Remark 3.22

To verify Assumption 3.21, it would suffice, by Lemma 3.13, that *P* is linear so that $\mu$ is Gaussian, or that an a priori estimate holds of the form

$$\begin{aligned} \Vert v_1\Vert _{\mathcal{C}^\gamma (\textbf{T}^d)} \lesssim (2+\Vert \boldsymbol{\Psi }\Vert _{\mathfrak {B}})^q \end{aligned}$$

*uniformly* over initial conditions $v_0$ (this is a strengthening of Assumption 3.19). This form of ‘coming down from infinity’ is known for the $\Phi ^4_2$ model [53, 59] and for more singular equations [24, 55]. Since this moment estimate is a separate task, we prefer to leave it as an assumption.

#### Lemma 3.23

Let $\phi \in \mathcal{C}^\infty (\textbf{T}^d)$ . Then, for every integer $k \ge 1$,

$$\begin{aligned}  &   {\textbf{E}}_{u_0\sim \mu } \Big [ k{\langle u_0, \phi \rangle }^{k-1}({\langle u_0, (\Delta -1)\phi \rangle } + {\langle \,:\!\! P(u_0) :\! ,\phi \rangle }) \\  &   \qquad + \frac{k(k-1)}{2} {\langle u_0, \phi \rangle }^{k-2} {\langle \textrm{Cov}_\xi *\phi , \phi \rangle } \Big ] = 0\;, \end{aligned}$$

where the final term in the expectation is zero if $k=1$.

#### Proof

This is identical to the proof of Lemma 2.5 upon substituting the use of Lemma 2.4 by Lemma 3.20. $\square$

#### Theorem 3.24

Suppose the degree *p* of *P* is odd and $p\ge 3$. Then $\mu$ is not Gaussian.

#### Proof

Following the proof of Theorem 2.7, and using the shorthand $X = {\langle u_0,\varphi \rangle }$, $Y = {\langle \,:\!\! P(u_0) :\!,\varphi \rangle }$, and $Z = {\langle u_0,(\Delta -1)\varphi \rangle }$ for $\phi \in \mathcal{C}^\infty (\textbf{T}^d)$, Lemma 3.20 implies the recursion ${\textbf{E}}{X^{k}(Y-Z)} = \frac{k}{2}{\textbf{E}}{X^{k-1}}$ for all $k\ge 0$, where the right-hand side is understood as zero for $k=0$.

Suppose now that $\mu$ is Gaussian. Similar to the the proof of Lemma 3.17, we write $u_0{\mathop {=}\limits ^{{\tiny law}}}u_1 = g + \Phi$ where $\Phi \sim \nu$ and $g\in \mathcal{C}^\gamma (\textbf{T}^d)$. Let us write $K(x,y) = {\textbf{E}}[\Phi (x)\Phi (y)]$. By Assumption 3.9 and Young’s convolution inequality, for $\frac{1}{r}+1 = \frac{1}{p} + \frac{1}{q}$,

$$\begin{aligned} \Big \Vert \int K(\cdot ,y)\phi (y)\,\textrm{d}y \Big \Vert _{L^r} \le \Big \Vert \int |\cdot -y|^\rho |\phi (y)| \,\textrm{d}y \Big \Vert _{L^r} \lesssim \Vert \phi \Vert _{L^q} \end{aligned}$$

where we used that $\rho p > -d$ so $\Vert |\cdot |^\rho \Vert _{L^p}<\infty$. Hence the convolution operator $K:L^q\rightarrow L^r$ is bounded. Taking $r=2p$, so that $\frac{1}{2p} + \frac{1}{q} = 1$, we obtain

$$\begin{aligned} {\textbf{E}}{\langle \Phi ,\phi \rangle }^2 = {\langle K\phi ,\phi \rangle } \lesssim \Vert \phi \Vert _{L^q}^2\;. \end{aligned}$$

Furthermore, by Assumption 3.19, ${\textbf{E}}\Vert g\Vert _{\mathcal{C}^\gamma }^q < \infty$ for all $q>0$. Therefore

$$\begin{aligned} |{\textbf{E}}{\langle \Phi ,\phi \rangle }{\langle g,\psi \rangle }| \le {\textbf{E}}[{\langle \Phi ,\phi \rangle }^2]^{1/2}{\textbf{E}}[{\langle g,\psi \rangle }^2]^{1/2} \lesssim \Vert \phi \Vert _{L^q}\Vert \psi \Vert _{L^1}\;, \end{aligned}$$

and ${\textbf{E}}{\langle g,\phi \rangle }^2 \lesssim \Vert \phi \Vert _{L^1}^2$. It readily follows that

$$\begin{aligned} |{\textbf{E}}{\langle u_0,\phi \rangle }{\langle u_0,\psi \rangle }| \lesssim \Vert \phi \Vert _{L^q}\Vert \psi \Vert _{L^q}\;. \end{aligned}$$

Hence, by duality, the covariance $C(x,y)= {\textbf{E}}[u_0(x)u_0(y)]$ defines via convolution a bounded operator $C:L^q \rightarrow L^{2p}$.

Using the notation $Q_k$ and $\hat{X}$ from Theorem 2.7, it follows from an identical argument that ${\textbf{E}}Q_k(\hat{X}) Y = 0$ for all $k\ge 2$. Recall also that $Q_k(\hat{X})$ has the Wiener-Itô decomposition $Q_k (\hat{X}) = I_k (\phi ^{\otimes k})$.

Using that $Y=\int _{\textbf{T}^d} \,:\!\! P(u_0) :\!(x) \phi (x)\,\textrm{d}x$, it follows from the Wiener–Itô decomposition of $\,:\!\! P(u_0) :\!$ at the top chaos^3^ that

$$\begin{aligned} 0 = {\textbf{E}}Q_p(\hat{X}) Y \propto \int _{\textbf{T}^d} (C\phi )(x)^{p} \phi (x) \,\textrm{d}x\;, \end{aligned}$$

where $p\ge 2$ is the degree of *P*. By considering a smooth approximation of a non-zero real eigenfunction of *C* with eigenvalue $\lambda > 0$ and using that $C:L^2\rightarrow L^{2p}$ is bounded, we obtain a contradiction in the same way as the end of the proof of Theorem 2.7. $\square$

#### Remark 3.25

Similar to Remark 2.9, our method of proof can handle more general polynomial non-linearities of the type (2.16). For example, it shows the invariant measure of

$$\begin{aligned} (\partial _t +1 -\Delta ) u = u^{:2:} - \Big (\int _{\textbf{T}^2} {u^{:2:}}\Big ) u + \xi \;, \qquad u_0\in \mathcal{C}^{\alpha }(\textbf{T}^2)\;, \end{aligned}$$

which is the $\Phi ^3_2$ measure

$$\begin{aligned} \mu (\,\textrm{d}u) = Z^{-1} \exp \Big ( -\frac{1}{3}\int _{\textbf{T}^2} {u^{:3:}} - \frac{1}{4}\Big (\int _{\textbf{T}^2} {u^{:2:}}\Big )^2 \Big ) \nu (\,\textrm{d}u)\;, \end{aligned}$$

is non-Gaussian.

Another SPDE that is covered by the Da Prato–Debussche argument is the dynamical Sine-Gordon model in two spatial dimensions, given by

3.21 $$\begin{aligned} (\partial _t +1 -\Delta ) u = \,:\!\! \sin (\beta u) :\! + \sqrt{2} \xi \;, \end{aligned}$$

where $\beta \in {\textbf{R}}$ with $\beta ^{2} < 4 \pi$ and $\xi$ is a space-time white noise on ${\textbf{R}}\times \textbf{T}^{2}$. Here the Wick trigonometrical functions are defined by setting

3.22 $$\begin{aligned} \,:\!\! \sin (\beta \bullet ) :\!  &   = \lim _{\varepsilon \downarrow 0} e^{\beta ^{2} {\textbf{E}}\Psi _{\varepsilon }^{2}(0)} \sin (\beta \bullet )\;,\nonumber \\ \,:\!\! \cos (\beta \bullet ) :\!  &   = \lim _{\varepsilon \downarrow 0} e^{\beta ^{2} {\textbf{E}}\Psi _{\varepsilon }^{2}(0)} \cos (\beta \bullet )\;, \end{aligned}$$

where $\Psi$ is the stationary-in-time solution to the linear equation.

Writing $u = \Psi + v$ as before, one can show (again, with the restriction $\beta ^{2} < 4 \pi$) the local well-posedness of the equation

3.23 $$\begin{aligned} (\partial _t - \Delta + 1) v = \sin ( \beta v) \,:\!\! \cos ( \beta \Psi ) :\! + \cos ( \beta v) \,:\!\! \sin (\beta \Psi ) :\! \end{aligned}$$

Local well-posedness for the dynamic (3.21) in a probabilistically strong sense was first studied in [46]. Since (3.21) is the Langevin dynamic for the Sine-Gordon Euclidean Quantum Field Theory, it would be interesting to see if the arguments of this article could be applied to show that any invariant measure of (3.21) is non-Gaussian. This seems plausible, but there are some differences in this model that would require modifications to the argument.

One difference is that $\,:\!\! \sin (\beta \Psi ) :\!$ does not have a “top level Wiener chaos” like the polynomial interactions do, but this by itself is not an obstruction since $\,:\!\! \sin (\beta \Psi ) :\!$ is in $L^{2}(\Omega ,\nu )$ with infinitely many chaos contributions. The tricky step seems to be ruling out that, if $\mu$ is an invariant measure of (3.21), then $\mu$ is not a Gaussian with a covariance that has a stronger blow-up than the covariance of the stationary distribution of $\Psi$. In the Wick power case, the existence of $\,:\!\! u^{2} :\!$ implies that the blow-ups of the covariance of $\mu$ and the covariance of $\Psi$ have to be comparable. However, the same argument does not work for $\,:\!\! \sin (\beta \Psi ) :\!$ since the renormalization in (3.22) is blowing up something going to 0 rather than cancelling out a divergence.

## Data Availability

No datasets were generated or analysed during the current study.

## Appendix A Da Prato–Debussche regime vs. absolute continuity

In this appendix, we show that the DPD trick, as described n Section 3.1, works in situations where the invariant measure of *u* is singular with respect to $\nu$, the Gaussian measure from Definition 3.4, which is invariant for the equation $(\partial _t +1-\Delta )\Psi = \xi$.

For simplicity, suppose $\textrm{Cov}_\xi = (1-\Delta )^{\beta }$ where $\beta \le 0$ and $\rho {\mathop {=}\limits ^{{\tiny def}}}2-d-2\beta \le 0$. (In particular, we suppose $d\ge 2$.) Then

$$\begin{aligned} |(1-\Delta )^{-1}*\textrm{Cov}_\xi (x)| = |(1-\Delta )^{-1+\beta }(x)| \lesssim {\left\{ \begin{array}{ll} 1+|\log |x| | & \text {if } \rho = 0 \\ |x|^\rho & \text {if } \rho <0 \end{array}\right. } \end{aligned}$$

(see, e.g. the proof of [23, Prop. A.1]). To apply the results of Section 3.1, due to Assumption 3.9, we require $\rho >2\alpha$ and $\rho > -d/p$ for some $\alpha <0$. Due to (3.5), we furthermore require $\alpha > 2/(1-2p)$. To ensure the existence of such $\alpha$, we thus need

A.1 $$\begin{aligned} \rho > \max \{4/(1-2p) , -d/p\}\;. \end{aligned}$$

On the other hand, a recent result of [45] is that $\textrm{Law}(u_t)$ for $t>0$ (and thus any invariant measure of *u*) is singular with respect to $\nu$. With the notation of [45, Sec. 3] on the left-hand side and ours on the right-hand side, we have $m=\beta$, $\sigma =2$, $k=p$, $n_i=0$, and $A = \rho p/2$. Therefore [45, Assumption 3.2] corresponds to $\rho <0$. Furthermore the first part of [45, Assumption 3.3] is equivalent to $\rho p + d + 2 > 0$, which is weaker than our assumption $\rho p + d>0$.^4^ The second part of [45, Assumption 3.3] is equivalent to $\rho p + d + 2 > -2\beta = \rho - 2 +d$, i.e. $\rho > -4/(p-1)$, which is again weaker than $\rho > 4/(1-2p)$.^5^ It is also simple to verify that (A.1) implies the final [45, Assumption 3.5].

It thus follows from [45, Thm 3.9] that $\textrm{Law}(u_t)$ is singular with respect to $\nu$ whenever (A.1) holds, $\rho <0$, and

A.2 $$\begin{aligned} \rho p + 2 \le -2\beta = \rho +d - 2\;. \end{aligned}$$

For $p > 1$, (A.2) is equivalent to

A.3 $$\begin{aligned} \rho \le \frac{d-4}{p -1} \end{aligned}$$

while for $p=1$, (A.2) is equivalent simply to $d\ge 4$.

We now claim that, for $p\ge 3$ and $d>2$, there exists $\rho < 0$ satisfying (A.1) and (A.3). (For $d=2$, the only possibility is $\rho =\beta =0$, in which case the result of [45] does not apply directly.) Indeed,

- for $d\ge 4$, clearly all $\rho \le 0$ satisfy (A.3), so the claim follows from the fact that the right-hand side of (A.1) is strictly negative,
- for $d=3$, (A.1) is equivalent to $\rho > 4/(1-2p)$ while (A.3) is equivalent to $\rho \le -1/(p-1)$, so the claim follows from the fact that $4/(1-2p) < -1/(p-1)$ for $p>\frac{3}{2}$.

### Example A.1

Take $p=3$ and $d=3$. Then, by (A.3), we have singularity of $\textrm{Law}(u_t)$ with respect to $\nu$ for $\rho \le -1/2$, i.e. $\alpha < -1/4$, which corresponds to the $\Phi ^4_{\delta }$ model with $\delta \ge \frac{5}{2}$ in the setting of [5, Sec. 2.8.2] (with $\textbf{T}^3$ taken therein instead of $\textbf{T}^4$). On the other hand, the DPD regime, by (A.1), requires only $\rho > -4/5$, i.e. $\alpha > -2/5$, which corresponds to the $\Phi ^4_{\delta }$ model with $\delta <\frac{14}{5}$.

## Footnotes

1 Essentially, $\textrm{Cov}_{\xi }$ should be no worse than a Dirac delta.

2 This in contrast to most other presentations which take the initial condition as 0 or take $\Psi$ as the stationary solution to $(\partial _t +1-\Delta )\Psi = \xi$, see e.g. [59].

3 Note that $\,:\!\! P(u_0) :\!$ is generally in the *inhomogeneous* *p*-th Wiener chaos of $\mu$ since the Wick ordering of $\,:\!\! P(u_0) :\!$ is with respect to $\nu$.
